# miR-181c Regulates the Mitochondrial Genome, Bioenergetics, and Propensity for Heart Failure *In Vivo*

**DOI:** 10.1371/journal.pone.0096820

**Published:** 2014-05-08

**Authors:** Samarjit Das, Djahida Bedja, Nathaniel Campbell, Brittany Dunkerly, Venugopal Chenna, Anirban Maitra, Charles Steenbergen

**Affiliations:** 1 Department of Pathology, Johns Hopkins University, Baltimore, Maryland, United States of America; 2 Department of Cardiology, Johns Hopkins University, Baltimore, Maryland, United States of America; 3 Australian School of Advanced Medicine, Macquarie University, Sydney, Australia; 4 Notre Dame of Maryland University, Baltimore, Maryland, United States of America; 5 Departments of Pathology and Translational Molecular Pathology, Houston, Texas, United States of America; 6 Department of Oncology, Johns Hopkins University, Baltimore, Maryland, United States of America; David Geffen School of Medicine at UCLA, United States of America

## Abstract

MicroRNAs (miRNAs) are small non-coding RNAs, which inhibit the stability and/or translation of a mRNA. miRNAs have been found to play a powerful role in various cardiovascular diseases. Recently, we have demonstrated that a microRNA (miR-181c) can be encoded in the nucleus, processed to the mature form in the cytosol, translocated into the mitochondria, and ultimately can regulate mitochondrial gene expression. However the *in vivo* impact of miR-181c is unknown. Here we report an *in-vivo* method for administration of miR-181c in rats, which leads to reduced exercise capacity and signs of heart failure, by targeting the 3′-end of mt-COX1 (cytochrome c oxidase subunit 1). We cloned miR-181c and packaged it in lipid-based nanoparticles for systemic delivery. The plasmid DNA complexed nanovector shows no apparent toxicity. We find that the mRNA levels of mitochondrial complex IV genes in the heart, but not any other mitochondrial genes, are significantly altered with miR-181c overexpression, suggesting selective mitochondrial complex IV remodeling due to miR-181c targeting mt-COX1. Isolated heart mitochondrial studies showed significantly altered O_2_-consumption, ROS production, matrix calcium, and mitochondrial membrane potential in miR-181c-treated animals. For the first time, this study shows that miRNA delivered to the heart *in-vivo* can lead to cardiac dysfunction by regulating mitochondrial genes.

## Introduction

Mitochondria, which contain their own DNA, mRNA, tRNA and ribosomes [1], are semi-autonomous organelles, but they import many critical proteins, which are encoded by nuclear genes [1], [2]. Critical to mitochondrial function are five respiratory chain complexes in the inner mitochondrial membrane that generate a proton gradient across the membrane, which produces ATP [1]–[4]. Most of the respiratory chain complex subunits are encoded by nuclear genes, except for some subunits of complex I, III, and IV, which are encoded by mitochondrial DNA and synthesized on mitochondrial ribosomes [1].

MicroRNAs (miRNAs) are small (∼19–22 nt), stable non-coding RNAs that critically modulate post-transcriptional gene regulation by binding at the 3′-UTR of corresponding mRNAs [5]. Because of this binding at the 3′-UTR of mRNAs, miRNA either block translation or cause message degradation through RNA Induced Silencing Complex (RISC) mediated events [5]. Several groups suggested that miRNA exist in mitochondria [6]–[10], but recent work from our group demonstrate that miRNA exist in heart mitochondria and are functionally important [11], [12]. We showed that miR-181c, derived from the nuclear genome, translocates to the mitochondria, and more importantly, regulates mitochondrial gene expression and affects mitochondrial function [11]. Coordination of nuclear gene expression and mitochondrial gene expression is thus essential. Our work shows that miRNA can regulate mitochondrial gene expression, specifically that miR-181c binds to the 3′-end of the mRNA of a mitochondrial gene, mt-COX1, a subunit of complex IV of the respiratory chain, and initially results in a decrease in mt-COX1 protein, complex IV remodeling, and increased production of reactive oxygen species [11].

In the present study, we examined whether this *in vitro* finding is applicable *in vivo*, using a novel miR-181c delivery system using lipid based cationic nanoparticles [13].

## Results

### *In vivo* characterization of nanovectors for systemic miR-181c delivery

**Figure 1:**
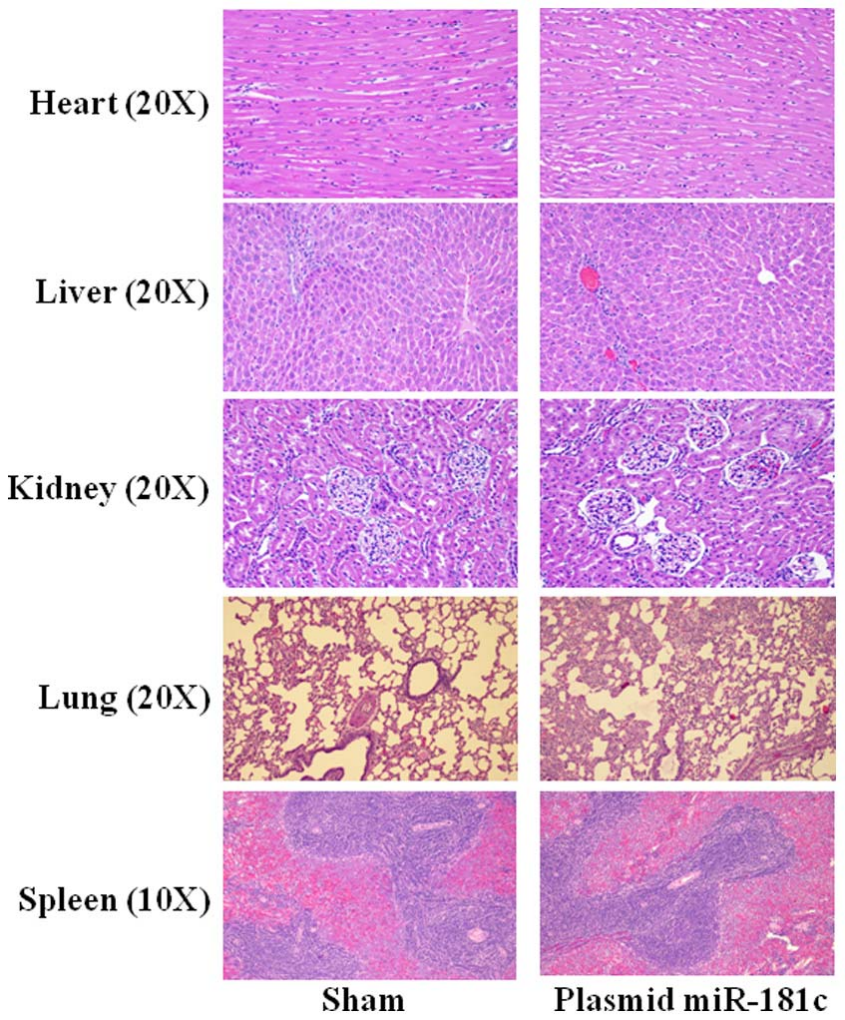
Effect of miR-181c in different organs by systemic delivery. 3 weeks after treating the rats with nanoparticles with/without miR-181c expression vector, we stained sections of five different organs with hematoxylin and eosin (H&E). Image magnification was 20× for heart, liver, kidney, and lung, and 10× for spleen.

**Figure 2:**
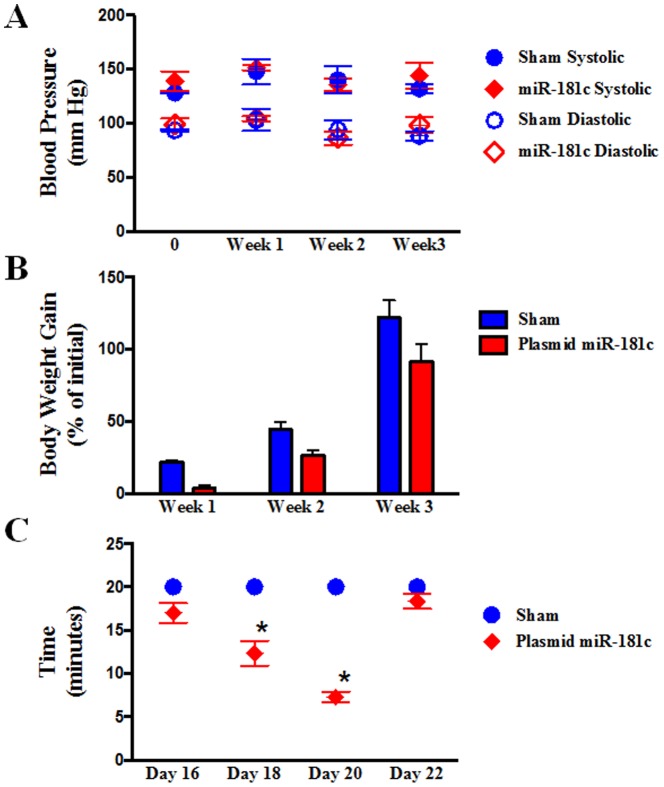
Physical Properties of the rats during the Treatment Protocols. (A) **Blood Pressure.** We used a non-invasive, tail cuff method, to measure diastolic blood pressure (DBP) and systolic blood pressure (SBP). We did not observe any significant difference between the two groups, either in DBP or SBP. The red color indicates the plasmid miR-181c treated group, and the blue color indicates the sham group. n = 3. (B) **Body weight**. Rats were weighed during the treatment period, and we found that the weight gain of miR-181c-treated rat group is significantly lower than the sham group, up to day 11. This is not a toxic reaction, but it is due to the high activity of these animals. These animals are not diabetic (dipstick and blood glucose tests were performed). But after day 13, miR-181c-treated animals become lethargic. The red bar indicates the plasmid miR-181c treated group, and the blue bar indicates the sham group. n = 12.(C) **Forced Swimming Test.** miR-181c overexpression shows a significant difference in exercise capacity compared to the sham group after the 18th day of treatment. Together with the echocardiography data we optimized day 20 for assessment.

The nanovector is an electrostatic complex of positively charged liposomal nanoparticles and negatively charged plasmid DNA (in this case, expressing miR-181c) [13]. After pilot *in vivo* studies using several doses and different time-points, we optimized a 4 mg/kg dose of plasmid DNA, which increases miR-181c levels in heart tissue ∼2 fold with 6 injections over 3 weeks. Thereafter, we evaluated the potential for adverse effects from systemic nanovector therapy by evaluating both hematologic and histologic samples (Fig. 1) from rats that had received either sham (empty vector) or miR-181c nanovector. No detectable amounts of serum pro-inflammatory cytokines, TNF-α, IL-2, or IFN-γ, were found in either group, 8 hours after I.V. administration. We also monitored the blood pressure of the animals during the treatment period, and did not observe any difference between the two groups (Fig. 2A). We observed that miR-181c overexpression groups are more physically active and gain less weight during the first 2 weeks of the treatment (Fig. 2B); we measured serum and urine glucose and found no evidence that the animals were diabetic. We also observed that the miR-181c-treated group develops fatigue much earlier during exercise than the sham group. Using a forced swimming test [14], we have found that the miR-181c-treated animals could only swim for 7–9 minutes on the 20th day of the treatment, whereas the sham group could swim the entire 20 minutes without any sign of fatigue (Fig. 2C).

### Systemic miR-181c delivery with nanovectors shows signs of heart failure

**Figure 3:**
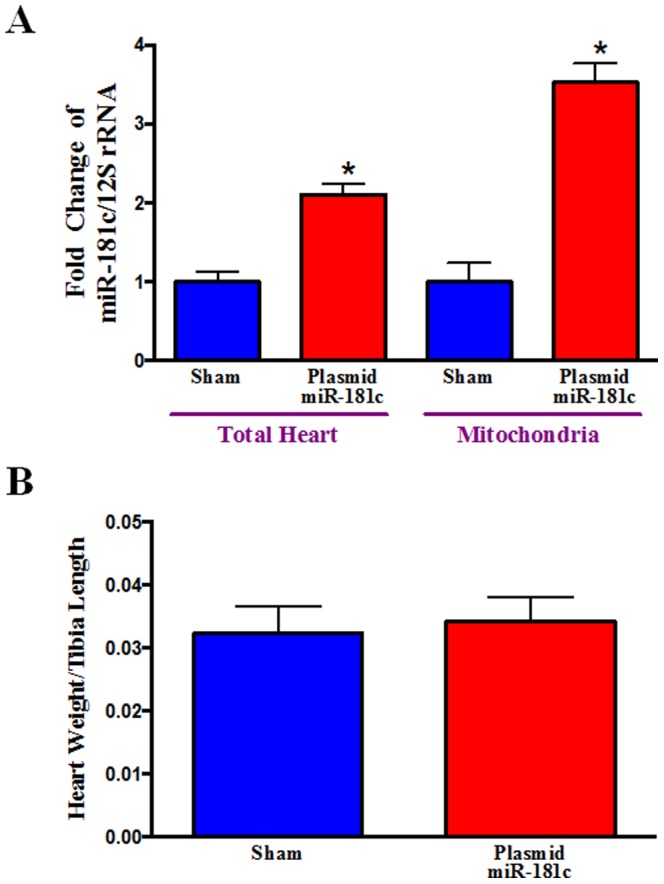
Effect of 3 weeks of nanovector delivery (i.v). (A) **miR-181c expression in the Heart and in the Heart-derived Mitochondrial Fraction.** qPCR shows that miR-181c expression in the whole heart homogenate is almost 1.5 times higher in the plasmid miR-181c group compared to its sham group. We isolated mitochondrial miRNA enriched total RNA, using our published protocol [5]. We also monitored the quality and integrity of the isolated RNAs, as described previously[5]. qPCR shows that miR-181c expression is almost 2.5 times higher in the miR-181c-treated group compared to its sham group. We used the mitochondrial gene product, 12S rRNA, as a normalization control [5]. *p<0.05 vs. sham (n = 6 for whole heart, and n = 4 for the mitochondrial fraction). (B) **Heart Weight.** We measured the wet weight of the whole heart and measured the corresponding tibial length of each rat. The ratio of heart weight to tibial length is not different among the groups, suggesting no sign of hypertrophy in the miR-181c-treated rats. (n = 12).

**Figure 4:**
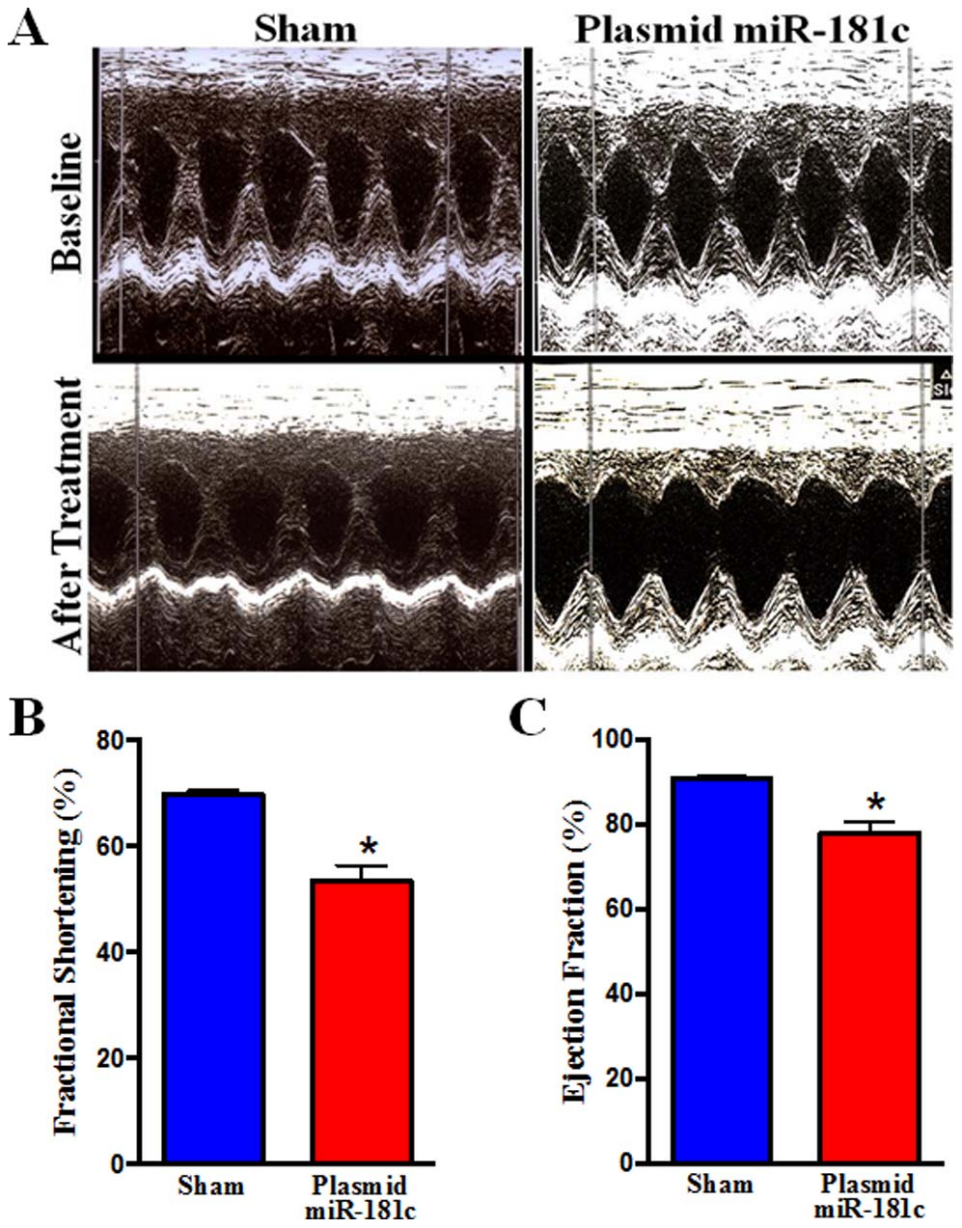
Echocardiography. (A) 2D M-mode and Doppler echocardiography was performed on non-anesthetized rats, before (top) and after (lower) the sham (left) or the miR-181c expression vector (right) treatment. (B) Percent fractional shortening, and (C) ejection fraction, were calculated using the software of the echocardiography instrument (n = 10).

We measured the expression of miR-181c in the heart, and found a ∼2 fold increase (Fig. 3A). We had previously found that endogenous miR-181c localizes to mitochondria [11], so we isolated RNA from the mitochondria [11] of the hearts treated with nanovector *in vivo*, and the miR-181c-treated group shows a significantly higher level of miR-181c in the heart-derived mitochondria (Fig. 3B). Although we did not find any sign of hypertrophy (Fig. 3C), using echocardiography, we find that miR-181c overexpression causes a significant decrease in left ventricular fractional shortening (FS) and markedly lower ejection fraction (EF) (Fig. 4A–C).

### Systemic miR-181c delivery target mitochondrial gene in the heart

**Figure 5:**
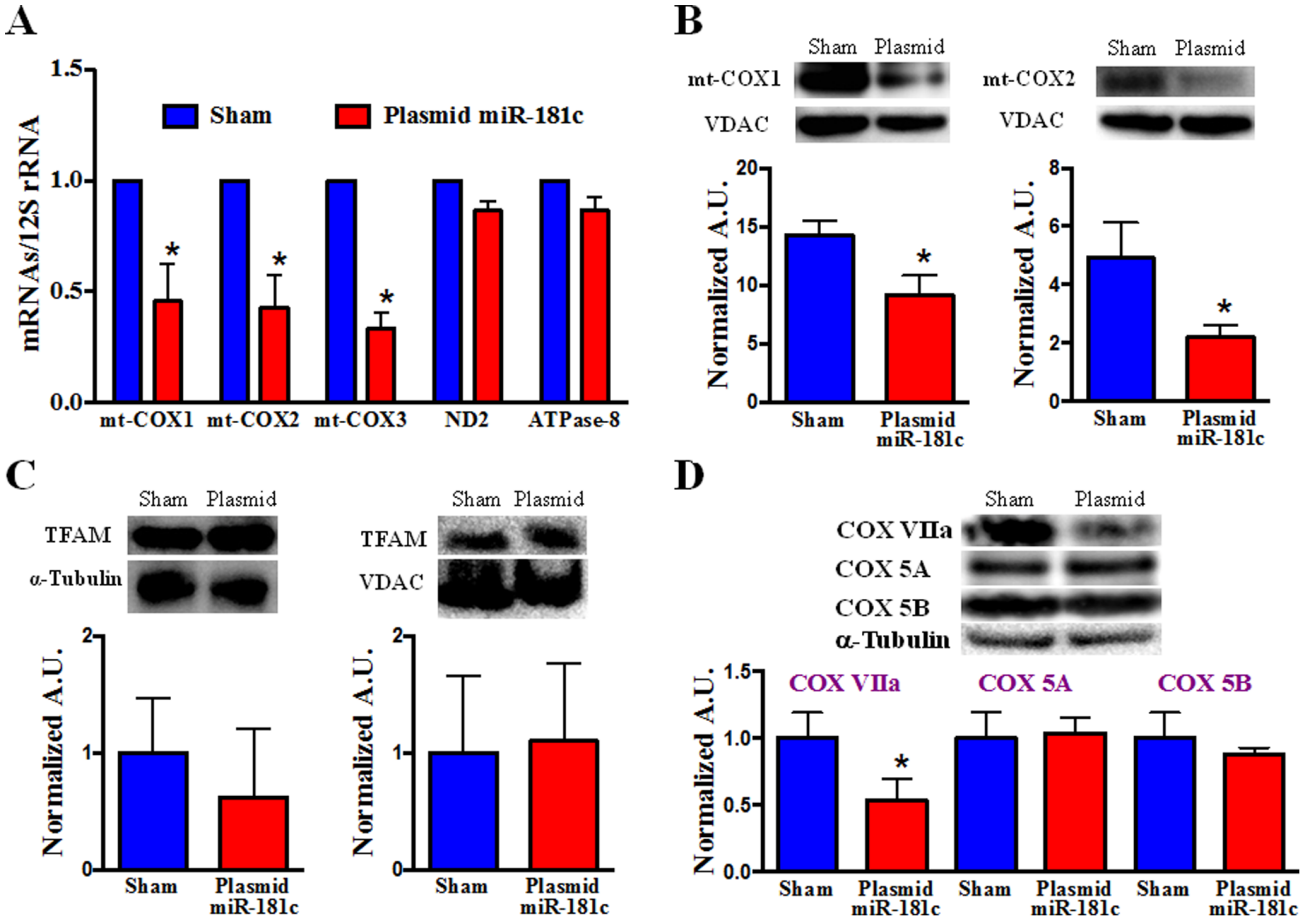
Mitochondrial Complex IV Remodeling after miR-181c Treatment. (A) qPCR data show that overexpression of miR-181c significantly reduces the mRNA levels of all mitochondrial complex IV genes with 3 weeks treatment. The treatment protocol has no effect on other mitochondrial genes, such as ND2 (complex I) and ATPase 8 (complex V). Content of mRNA was first normalized to 12S rRNA, a mitochondrial gene, as 12S rRNA expression did not change with miR-181c overexpresssion. Then we normalized the data to the sham group. *p<0.05 vs. sham (n = 6). (B) Western blot shows that miR-181c overexpression significantly reduces the protein content of both mt-COX1 and mt-COX2. VDAC was used as a loading control. The data were normalized to the sham group. *p<0.05 vs. sham (n = 6). (C) Western blot shows that miR-181c overexpression has no effect on Transcription Factor A, Mitochondria (TFAM), either in the total heart homogenate (left panel) or the heart-derived mitochondrial fraction (right panel). TFAM plays an important role in mitochondrial gene transcription, by activating 3 different promoter regions in the D-loop area of the mitochondrial genome. TFAM also translocates from the cytosol to the mitochondria as part of the mitochondrial gene transcription process. α-tubulin (for total heart homogenate) and VDAC (mitochondrial fraction) were used as loading controls. The data were normalized to the sham group (n = 3). (D) Western blot shows the changes of other isoforms of mitochondrial respiratory chain complex IV. We have observed a significant decrease in the protein content of COX VIIa in the miR-181c overexpression groups, but no effect on COX 5A and COX 5B. α-tubulin was used as the loading control. The data were normalized to the sham group (n = 3).

We next assessed the functional consequences of translocation of miR-181c into the mitochondria. We have previously found that miR-181c can regulate the mt-COX1 gene and causes mitochondrial complex IV remodeling [11]; therefore, we measured each of the mitochondrial genome components of complex IV. Indeed, we found a significant decrease of both mRNA (Fig. 5A) and total protein content (Fig. 5B) of mt-COX1 in the miR-181c overexpressing hearts, confirming our *in-vitro* observation that miR-181c regulates mt-COX1 expression [5]. We also observed a significant decrease in mRNA levels of both mt-COX2 and mt-COX3 but not any other mitochondrial genes, such as complex I or V components, ND1 or ATPase 8 (Fig. 5A); there was also no effect on TFAM level (Fig. 5C). This further confirms our *in-vitro* observation of mitochondrial complex IV remodeling [11]. Unlike our acute *in-vitro* study, at Day 20 of the nanovector treatment, we found a significant decrease of mt-COX2 protein (Fig. 5B), suggesting that a decrease in the expression of mt-COX1 over time results in a reduction in the amount of other Complex IV components. This differs from our earlier *in-vitro* [11] work, in which, after 48 hr of miR-181c overexpression, we observed a significant decrease in mt-COX1 protein levels, but a significant increase in mt-COX2 protein levels. This likely reflects the longer duration of miR-181c overexpression in our *in vivo* protocol. We suggested that complex IV remodeling was occurring and that as a result of lower mt-COX1 levels, an adaptive response was activated, resulting in higher mt-COX2 levels. Unlike our *in-vitro* data, this *in vivo* (day 22) data suggests that miR-181c significantly decreases the mRNA and protein content of multiple complex IV mitochondrial genes (mt-COX1, mt-COX2 and mt-COX3) (Fig. 5A, 5B). Figure S1 in File S1 shows that a shorter (2 week) treatment causes changes in mRNA expression levels of mitochondrial complex IV subunits in a manner that more closely resembles what we have observed *in-vitro*. *In vivo*, we did not see a significant change either in miR-181c expression (Fig. S2 in File S1), or in mRNA levels of mt-COX1 or mt-COX2 in the heart at 2 weeks; however, we did observe a significantly higher level of mt-COX3. These data suggest that miR-181c overexpression *in vivo* has progressive effects on mitochondrial complex IV. It appears that only complex IV is altered since we have measured ND2 and ATPase 8 mRNA levels and have not found any changes with miR-181c overexpression (Fig. S1 in File S1). However, changes occur not just in COX subunits that are products of the mitochondrial genome, but also in COX subunits that are derived from the nuclear genome such as COX VIIa (Fig. 5D). On the other hand, not all COX subunits are downregulated. We found that COX 5A and COX 5B are not significantly altered by chronic miR-181c overexpression *in vivo*. The effect of miR-181c overexpression using nanovector delivery is transient. We observed that miR-181c expression was not increased in several animals that were followed for 3 weeks after the completion of treatment, and the transient nature of miR overexpression with nanovector treatment has been reported previously [13].

### Systemic miR-181c delivery regulates Mitochondrial Function

**Figure 6:**
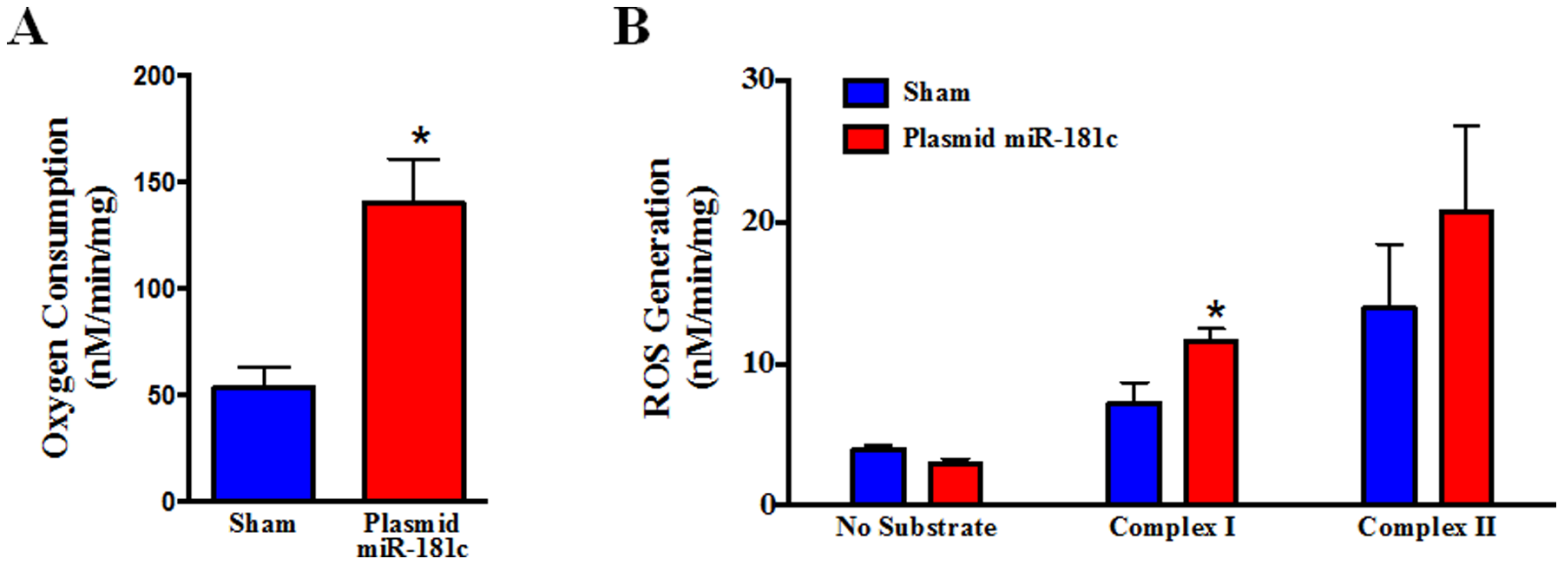
Mitochondrial function. Effect of 3 weeks of nanovector treatment on isolated mitochondria. (A) O_2_ consumption was measured after adding the substrate for complex IV, TMPD/ascorbate. Overexpression of miR-181c markedly increases respiration compared to the sham, after adding TMPD/Ascorbate. These data show the role of miR-181c on mitochondrial energy metabolism by altering complex IV. *P<0.05 vs sham, n = 6. (B) Rate of ROS generation from heart-mitochondria overexpressing miR-181c. Without any substrate the rate of ROS production is not different between the two treatment groups. But, using glutamate/malate (complex I) and succinate (complex II), ROS production is significantly higher in the miR-181c overexpression groups. *p<0.05 vs. sham, n = 4.

To determine whether miR-181c regulates mitochondrial energy metabolism, we measured O_2_ consumption [15]. To focus specifically on complex IV, we used the complex IV substrates, TMPD and ascorbate. Complete inhibition of Complex IV would lead to metabolic inhibition and loss of oxidative phosphorylation (OXPHOS) as a means of energy generation. However, partial inhibition through reduced expression of Complex IV subunits has a more complex effect on mitochondrial function. As we saw in our *in-vitro* study [11], the rate of O_2_ consumption is significantly increased in miR-181c overexpressing mitochondria compared to the sham after adding complex IV substrate (Fig. 6A). To further evaluate the mechanism by which miR-181c alters mitochondrial respiration, we measured ROS production in isolated mitochondria. Overexpression of miR-181c significantly increased the rate of ROS generation in the miR-181c-treated group (Fig. 6B). We used two different substrates, glutamate/malate for complex I and succinate for complex II. We could not use TMPD/ascorbate because TMPD interferes with the amplex red signal. The increase in ROS production in isolated heart mitochondria from the miR-181c-treated group provides a partial explanation for the increase in O_2_ consumption.

**Figure 7:**
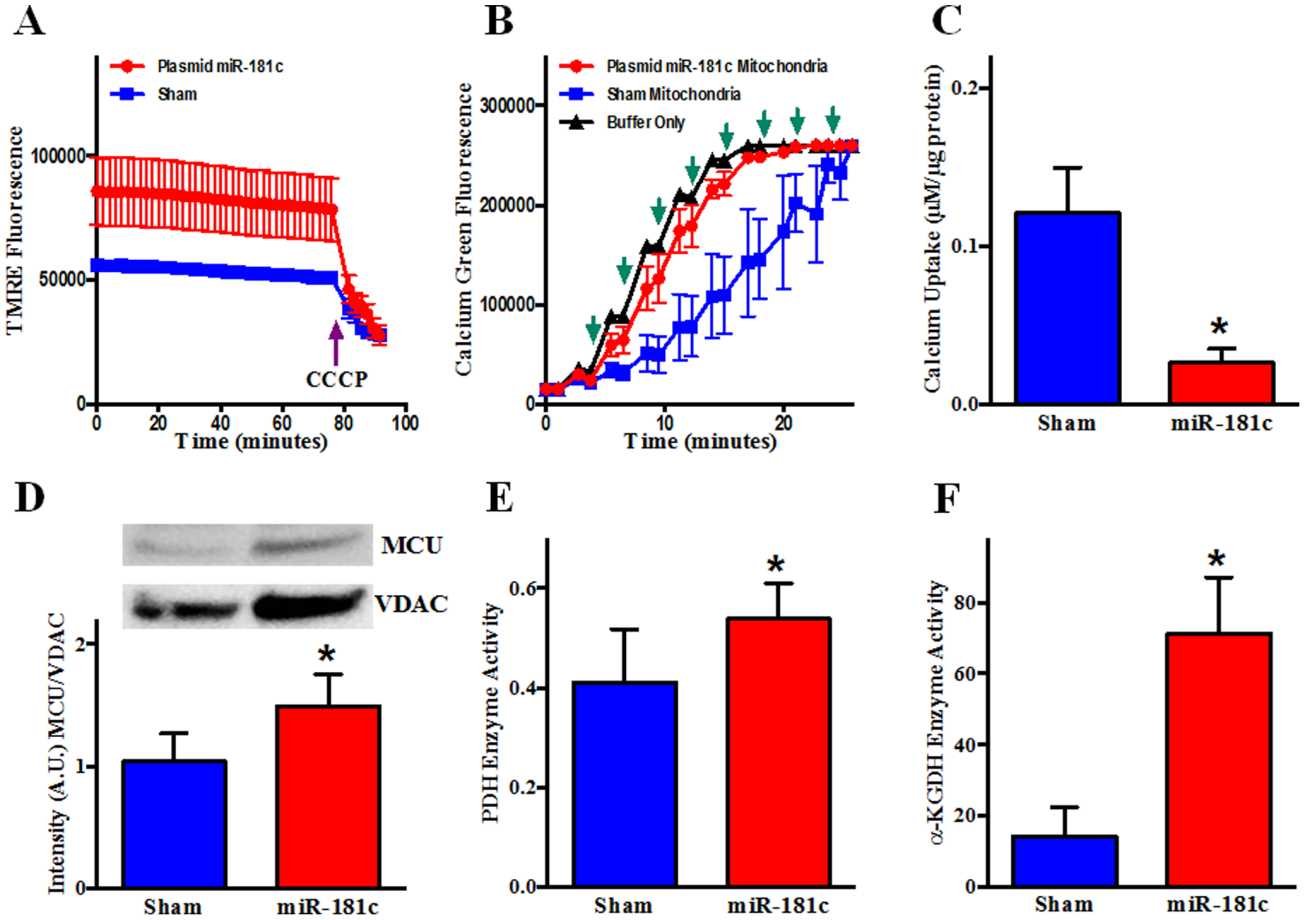
Effect of miR-181c on Mitochondrial Function. Heart-derived mitochondria were isolated from the two groups of rats after 3 weeks of treatment. (A) TMRE (tetramethylrhodamine methyl ester) fluorescence was measured from mitochondria supplied with glutamate/malate from two different treatment groups for 25 min, followed by the addition of the uncoupler, CCCP (Carbonyl cyanide m-chlorophenyl hydrazone). The miR-181c treatment group showed a higher level of TMRE intensity before the CCCP addition. n = 3. (B) Isolated mitochondria were incubated with Calcium Green 5N and fluorescence intensity was monitored after adding Ca^2+^. Following a Ca^2+^ pulse, fluorescence increases initially, but reverts back towards baseline as mitochondria take up Ca^2+^. When mitochondria take up enough Ca^2+^, the mPTP opens. This results in Ca^2+^ release and a decrease in fluorescence. (↑) indicates addition of 10 µM Ca^2+^. n = 3. (C) Western blot shows that miR-181c overexpression significantly increases the protein content of the mitochondrial calcium uniporter (MCU) both from total heart homogenate and mitochondrial fraction. VDAC was used as a loading control. The data were normalized to the sham group. *p<0.05 vs. sham (n = 4). (D) pyruvate dehydrogenase (PDH) and (E) α-ketoglutarate dehydrogenase (αKGDH) activities were significantly higher in the isolated mitochondria from the miR-181c treated hearts compared to sham, suggesting that the mitochondrial matrix Ca^2+^ concentration is increased in the miR-181c treated animals.

To further characterize the effect of miR-181c overexpression on mitochondrial function, we measured mitochondrial membrane potential (ΔΨ_m_). We used TMRE [16], [17] to measure ΔΨ_m_ in isolated mitochondria and found that the miR-181c-treated group shows a markedly higher level of TMRE fluorescence compared to the sham group, suggesting a significantly higher ΔΨ_m_ with miR-181c overexpression (Fig 7A). ΔΨ_m_ dissipates completely upon addition of 20 µM CCCP, as is observed in the end of the TMRE fluorescence graph in Figure 7A.

One explanation for this high ΔΨ_m_ would be if miR-181c overexpression causes mitochondrial matrix Ca^2+^ concentration to increase [18], [19] and this activates calcium-sensitive mitochondrial dehydrogenases [19], [20]. To assess whether mitochondrial matrix Ca^2+^ concentration is increased, we examined direct targets of Ca^2+^ in the mitochondrial matrix such as α-ketoglutarate dehydrogenase (αKGDH) and pyruvate dehydrogenase (PDH), which is regulated by a calcium-sensitive dephosphorylation mechanism. Activity of both of αKGDH and PDH are increased in the presence of calcium and we measured an increase in activity of both PDH (Fig. 7E) and αKGDH (Fig. 7F) in the miR-181c-treated mitochondria. To investigate possible mechanisms to explain the increase in matrix calcium, we examined the expression of the mitochondrial calcium uniporter (MCU), and found that MCU expression was increased in the miR-181c treatment group (Fig. 7D). Although the miR-181c-treated mitochondria are fully energized, an increase in matrix Ca^2+^ may have detrimental effects on the ability of mitochondria to take up and retain additional Ca^2+^, and may make the mitochondria more susceptible to opening of the mitochondrial permeability transition pore. To assess the mitochondrial calcium retention capacity, Ca^2+^ was added to mitochondria in small increments. As shown in Figure 7B-C, mitochondria derived from miR-181c-treated hearts have a reduced ability to accumulate and retain Ca^2+^ and are very susceptible to mPTP opening with Ca^2+^ addition. After addition of sufficient calcium to complex the EGTA in the buffer (40 µM), we added 10 µM Ca^2+^ every 2 min. After only 2 boluses of Ca^2+^, the mitochondria released the accumulated Ca^2+^ in the miR-181c overexpression group, indicating mPTP opening, whereas it took the sham group 8 additional Ca^2+^ pulses before Ca^2+^ release occurred. Thus there are potential detrimental effects of the increase in matrix calcium, and the increase in ROS, that occurs with miR-181c overexpression.

## Discussion

Over the past few years, there have been numerous studies that have pointed to the significant role of miRNAs in heart diseases by regulating nuclear genes [21], [22]. We have shown that miRNA can also target the mitochondrial genome in myocytes [11]. There are multiple novel aspects of this study, which further reveal mechanistic insights concerning the pathophysiologic effects of prolonged miR-181c overexpression, involving mitochondrial gene regulation, *in-vivo*.

To our knowledge, this is the first study to address the functional consequences of a MitomiR (viz., miR-181c) in the heart and its physiologic relevance *in-vivo*. Secondly, although nanovector delivery of a miRNA is a well established approach in oncology, our study is the first demonstration that a miRNA can be manipulated in non-neoplastic tissue using a plasmid-containing nanovector. We find that reduced expression of mitochondrial complex IV subunits leads to increased matrix calcium and activation of citric acid cycle dehydrogenases that increase the flow of protons and electrons into the electron transport chain, resulting in an increase in ΔΨ_m_, while there is a decrease in complex IV activity, which shunts electrons into alternate pathways, culminating in increased ROS production. The high ΔΨ_m_ indicates that mitochondrial function is preserved and provides a thermodynamic basis for high matrix [Ca^2+^]. Thus despite reduced activity of complex IV, respiration is overall higher in the miR-181c-treated group (Fig 6A).

One of the key findings of this study is that overexpression of miR-181c leads to dysfunction of complex IV, which activates ROS generation and O_2_ consumption; involving increased matrix [Ca^2+^]. Increased matrix [Ca^2+^] activates Complex III and several dehydrogenases, leading to an increase in ΔΨ_m_ [18], [19]. Concurrently there is an increase in ROS generation, perhaps related to both the increase in membrane potential and the increase in citric acid cycle activity, while the transfer of electrons and protons to molecular oxygen through Complex IV is inhibited. Despite the increase in ROS generation, ΔΨ_m_ remains high and the mitochondria are fully energized. There are numerous studies explaining the potential role of complex III in ROS generation [1], and there is also abundant evidence that complex I can generate ROS, particularly in the context of high ΔΨ_m_ and partial inhibition of complex IV [1]. Others have found that partial inhibition of complex IV can markedly increase ROS production without necessarily decreasing ATP synthesis [23]. An increase in matrix Ca^2+^ concentration, by increasing the activity of several citric acid cycle dehydrogenases [19], [20], would increase the flow of electrons and protons into the electron transport chain, increasing ΔΨ_m_ [18], [19]. ΔΨ_m_ is the driving force for Ca^2+^ uptake [23], and furthermore, this increase in ΔΨ_m_ could explain the increase in ROS, as the flow of electrons and protons into the electron transport chain increases while the terminal step is partially inhibited. This study highlights an unusual aspect of mitochondrial biology, involving complex IV remodeling and ROS generation.

The heart gets most of its energy from electron transport, coupled to oxidative phosphorylation. O_2_ is the ultimate electron acceptor for the mitochondrial respiratory chain, and the addition of electrons and protons to O_2_ to form water is catalyzed by cytochrome c oxidase, the terminal complex in the respiratory chain. mt-COX1 is a core component of cytochrome oxidase in the inner membrane of the mitochondria. Loss of cytochrome oxidase activity would be expected to affect mitochondrial energy metabolism, and mitochondrial dysfunction is important in many diseases [24]–[27]. Even though complex IV plays a critical role in the electron transport chain and in OXPHOS, the consequences of altered complex IV function are poorly understood. In a yeast model, it has been shown that mt-COX1 binds to mt-COX2 with the help of Mg^2+^ or Mn^2+^ ions, and this forms the catalytic core of complex IV [28]. In this study we have shown that chronic overexpression of miR-181c in the rat heart leads to significant down-regulation of mt-COX1 and mt-COX2 (Fig. 5A–B). Despite complex IV remodeling and reduction in the expression of key subunits following miR-181c overexpression, overall mitochondrial function and membrane potential are well maintained because of an increase in matrix [Ca^2+^], which increases the activity of other electron transport components. Although mitochondrial function is maintained, the high membrane potential and partial inhibition of complex IV leads to increased ROS production. Increased ROS production could eventually lead to impaired mitochondrial function which ultimately could be associated with decreased ΔΨ_m_, and ROS production in the setting of high matrix [Ca^2+^] could lead to opening of a non-specific pore in the mitochondrial membrane known as the mitochondrial permeability transition pore (mPTP) [3], [4], [15]. Increased ROS production could have other consequences as well, including reduced contractile function, as has been observed with postischemic stunning [31], [32]. In fact, like our miR-181c overexpressor model, the role of ROS and Ca^2+^ in the pathogenesis of myocardial stunning is well documented [31], [33]; including mitochondrial matrix [Ca^2+^] overload [34].

Our data suggest that complex IV remodeling affects more than just the subunits that are products of the mitochondrial genome and subject to direct regulation by miR-181c within the mitochondrial matrix. There are also changes to peripheral complex IV subunits that are products of the nuclear genome such as COX VIIa, but not to all of the subunits derived from the nuclear genome such as COX 5A and COX 5B. The loss of COX VIIa protein over time, as well as mt-COX2, may be related to increased degradation. A role for LON-mediated degradation of COX subunits in regulation of complex IV subunit composition has been reported previously [35].

Taken together, the results demonstrate that chronic overexpression of miR-181c has a role in heart failure by targeting the mitochondrial gene, mt-COX1, which ultimately leads to dysfunctional complex IV, altered mitochondrial metabolism, and ROS generation. We have also identified an underlying mechanism by which complex IV inhibition can activate ROS production in the mitochondria, by increasing matrix [Ca^2+^], which activates mitochondrial dehydrogenases. Our study used a novel systemic miRNA delivery system, using cationic nanovectors, in the heart. This technology may lead to innovative new therapeutic interventions for heart disease.

## Materials And Methods

### Animals

Male Sprague-Dawley rats (150–175 g, Harlan Sprague-Dawley) were used in this study. They were provided with food and water *ad libitum*. We tested glucose levels in both the blood and urine, using a blood glucose meter (OneTouch Basic, Milpitas, CA) and dipsticks. Rats were treated humanely and all experimental procedures were approved by the Institutional Animal Care and Use Committee of Johns Hopkins University.

### miR-181c expression constructs

miR-181c and 361 bp of flanking sequence was amplified from rat genomic DNA using polymerase, and cloned into the EcoRI and XhoI sites of the MSCV-Neo vector (Clontech Laboratories). The sequences of the amplified products were confirmed by both PCR and sequencing [29], [30]. Primer sequences are provided in Table 1.

**Table 1:** Primer Sequences for PCR and Sequencing.

Forward:	5′- TCTCCTGGGTGTCCAAAAAG-3′
Reverse:	5′- ACCCACCGACAACAATGAAT -3′

a) miR-181c.

### Preparation of nanovector for systemic miR-181c delivery in the rats

Liposomal nanoparticles were prepared by dissolving cationic amphiphile (DOTAP) and co-lipids (cholesterol and DSPE-PEG-OMe) in a 5∶5∶0.1 mM ratio, respectively, in a mixture of chloroform and methanol. The organic solvent was removed at 44–45°C, under vacuum by using a rotary evaporator, followed by a gentle flow of moisture-free nitrogen and the remaining dried film of lipid was then kept under high vacuum for 8 hours. 5% glucose was added to the vacuum-dried lipid film and the mixture was allowed to hydrate overnight. The vial was vortexed for 2 to 3 minutes at room temperature and occasionally shaken in a 45°C water bath to produce multilamellar vesicles. Small unilamellar vesicles were prepared by sonication of the multilamellar vesicles in an ice bath for 3 to 4 minutes until clarity, using a Branson 450 sonifier (Danbury) at 100% duty cycle and 25-W output power. The nanovector is an electrostatic complex of positively-charged liposomal nanoparticles and negatively-charged plasmid Nucleotide, and was prepared by mixing pMSCV-Neo vectors expressing miR-181c and liposome on a 1∶3 Nucleotide/lipid charge ratio basis. This nanovector is administered i.v. to rats through the tail vein, as described previously [13], [29].

### Experimental Protocol

We used a dose of 4 mg of nanovector/Kg body weight, and have optimized a regimen of 6 injections into the tail vein over 2 weeks. There is also a 1 week period (days 15–21) to allow expression of the vector *in vivo*. We optimized this protocol by monitoring cardiac function by echocardiography and forced swimming before, during, and after nanovector delivery.

### Echocardiography

Sequoia C256 ultrasound system, (Siemens, Mountain View, CA) equipped with 15-MHz linear array transducer was used. 2 Dimensional, M-mode, and Doppler echocardiography were performed to assess cardiac function and morphology in treated and sham treated rats, without anaesthesia [30].

### Forced Swim Test

The Forced Swim test or Porsolt swim test is a standard assay to assess exercise tolerance in rats [14]. Rats were placed in a cylinder (20 cm diameter, 45 cm height) filled with 25°C water to a level of 40 cm. Rats were monitored for changes in exercise tolerance after nanovector treatment for 2 weeks, with iv injection 3 times a week and then evaluated on day 16, 18, 20, and 22 following initiation of the treatment protocol. The rats remain in the water for a maximum of 20 minutes. The swim test was terminated if the rat showed signs of exhaustion. All sessions are videotaped and scored offline for time spent swimming. After each session, rats were removed and placed in a clean cage under a heating lamp to dry before being returned to the home cage or performing echocardiography [31].

### Langendorff Rat Heart Preparation

After sufficient anesthesia was achieved with ketamine (19 mg/Kg body wt.) - Xylazine (10 mg/Kg body wt.) cocktail, i.p., rats were anticoagulated with heparin sodium (500 IU/kg body weight, i.v. injection) (Elkin-Sinn Inc., Cherry Hill, NJ). Hearts were excised, cannulated, and perfused with Krebs-Henseleit buffer containing (in mmol/L) NaCl 120, KCl 5.9, MgSO_4_ 1.2, CaCl_2_ 1.25, NaHCO_3_ 25, and glucose 11. The buffer was aerated with 95% O_2_ and 5% CO_2_, to give a pH of 7.4 at 37°C as described previously [15]. All hearts are perfused to wash out blood and stabilize for 15 minutes, followed by perfusion with RNAlater (Qiagen, Valencia, CA), 10 ml diluted in Krebs-Henseleit buffer, for another minute [11].

### Isolated Mitochondria Protocols

Freshly isolated mitochondria were prepared from hearts after perfusion with RNAlater, by differential centrifugation [11]. Briefly, at the end of perfusion, the left ventricle was dissected out and placed in Buffer A (in mM: 180 KCl, 2 EGTA, 5 MOPS; 0.2% BSA; pH: 7.25). The tissue was then digested with trypsin (0.0001 g/0.1 g tissue) in 0.7 ml of ice-cold Buffer B (in mM: 225 Mannitol, 75 sucrose, 5 MOPS, 0.5 EGTA, 2 Taurine; pH: 7.25) and finally homogenized with Buffer B with a protease inhibitor cocktail (Roche Applied Science, Indianapolis, IN) using a Polytron. To further separate the heart mitochondria from other cellular components and tissue debris, a series of differential centrifugations were performed in a Microfuge 22R centrifuge (Beckman Coulter, Fullerton, CA) at 4°C. The crude pellet was then lysed with QIAzol (Qiagen, Valencia, CA) [11].

### RNA isolation

Total RNA were isolated, from whole hearts, mitochondrial fraction of the hearts, as described above, using a miRNeasy kit (Qiagen, Valencia, CA) and RNase free DNase kit (Qiagen, Valencia, CA) [11]. To characterize the integrity of the isolated RNA, spectrophotometric evaluation was performed, using Nanodrop (Thermo Scientific, Wilmington, DE). All the samples whose A_260_ (absorbance at 260 nm) value is more than 0.15 is used for further experiments. As the A_260_<0.15 suggest poor quality of RNA. The ratio of the readings at 260 nm and 280 nm (A_260_/A_280_) was also measured in order to check the purity of the isolated RNA.

### qRT-PCR

After performing the purity and integrity test, the RNA was reverse transcribed using a miScript Reverse Transcription Kit (Qiagen, Valencia, CA). PCR was performed using a miScript SYBR green PCR kit (Qiagen, Valencia, CA) and detected with a CFX96 detector (Bio-Rad, Hercules, CA). The primer sequences have been previously described [7]. All reactions were performed in triplicate.

### Mitochondrial Respiration Assay

The ADP-dependence of mitochondrial respiration was assessed at 25°C in a chamber containing respiration buffer (in mM) KCl 140, EGTA 10, HEPES 20, Oxalic Acid 5, K2HPO4 5 and pH 7.25 and connected with a Clark-type O2 electrode (Instech) and O2 monitor (Model 5300, YSI, Inc) [11]. After injecting the mitochondria (200 µg) into the air sealed chamber, Complex IV activity was measured by addition of TMPD/Ascorbate (0.2 mM and 5 mM) and the respiratory rate was determined [11].

### Reactive Oxygen Species (ROS) Production Assay

Hydrogen peroxide (H_2_O_2_) production from isolated mitochondria was measured fluorimetrically by measurement of oxidation of Amplex Red to fluorescent resorufin (Life Technologies, Carlsbad, CA). Isolated heart-mitochondria was incubated in buffer containing 120 mM KCl, 1 mM EGTA, 5 mM MOPS, and 5 mM K_2_HPO_4_ (pH 7.25). All incubations also contained 50 µM Amplex Red and 5 U/ml of horseradish peroxidase. The increase in fluorescence at an excitation of 544 nm and an emission of 590 nm was monitored. Standard curves were generated using known amounts of hydrogen peroxide [11].

### Mitochondrial Calcium Retention Capacity Assays

After the 3 week nanovector treatment protocol, we isolated the heart mitochondria, and the extra-mitochondrial calcium concentration was measured at room temperature in Costar 96-well plate reader by Fluostar (BMG Labtech Inc., Durham, NC) using 2 µM fluorescent Ca^2+^ indicator Calcium Green-5N (Molecular Probes, Eugene, OR). The fluorescence was excited at 485 nm and recorded at 520 nm. The concentration of isolated heart-mitochondria was 250 µg in a buffer containing 125 mM KCl, 10 mM MOPS, 1 mM KH_2_PO_4_, 2.5 mM MgCl_2_, and 20 µM EGTA; the pH was adjusted at 7.4. We added sufficient Ca^2+^ to chelate the EGTA, followed by 10 µM Ca^2+^ pulses to assess calcium retention capacity [17]. Two measurements were made after each pulse to demonstrate that a stable value had been obtained. In the buffer only group, there are no mitochondria and therefore the difference in fluorescence is a measure of how much Ca^2+^ was taken up by the mitochondria.

### Whole Hearts or Isolated Mitochondrial Fraction Preparation for Western Blot

Whole heart or isolated mitochondrial samples were lysed with RIPA buffer and protein content was measured using a Bradford assay [6]. Protein samples and molecular weight standards were separated by 1D gel electrophoresis. After transfer to a PVDF membrane, the membrane was incubated with antibody that recognizes proteins such as mt-COX-1 (Cat#sc-58347) (Santa Cruz Biotechnologies Inc., Santa Cruz), mt-COX2 (Cat#A-6404) (Life Technologies, Carlsbad, CA), MCU (Cat#ab121499), TFAM (Cat#ab131607), COX 5A (Cat#ab180129), COX 5B (Cat#ab110263) and COX VIIa (Cat#ab110268) (Abcam, Cambridge, MA) and VDAC (Cat#4866) (Cell Signaling Technologies, Danvers, MA) in Tris-Buffered Saline (pH 7.4) with 1% TWEEN 20 (TBS-T) with 5% BSA or nonfat dry milk at 4°C overnight. Membranes were incubated with the secondary antibody, appropriate horseradish peroxidase–conjugated IgG in TBS-T with 5% nonfat dry milk for 1 hour at room temperature. Immunoreactive protein was visualized using an enhanced chemiluminescence analysis kit (GE HealthCare, Piscataway, NJ).

### Statistics

All the data are presented as Means +SEM. Statistical significance (p<0.05) was determined between groups using ANOVA for multiple groups or Student *t*-test for two groups.

## Supporting Information

File S1 (DOC)
