# Supplementary material for: miR-181c Regulates the Mitochondrial Genome, Bioenergetics, and Propensity for Heart Failure In Vivo
Source: PLoS One. 2014 May 8;9(5):e96820. doi: 10.1371/journal.pone.0096820 (PMC4014556; doi:10.1371/journal.pone.0096820)
Supplement: File S1 — (DOC) [file pone.0096820.s001.doc]

**^1^S; Supporting Information for: miR-181c Regulates the Mitochondrial Genome, Bioenergetics, and Propensity for Heart Failure *in vivo***

**Das, Bedja, Campbell, Dunkerly, Chenna, Maitra and Steenbergen**

**Figure S1. Complex IV Remodeling with miR-181c overexpression.** qPCR data show that overexpression of miR-181c *in vivo* significantly reduces the mRNA levels of all mitochondrial complex IV genes, with 3 weeks treatment. But with shorter treatment (2 weeks), mt-COX2 (p=0.09) and mt-COX3 (p=0.04) expression notably increased, with no change of mt-COX1 (p=0.37) expression. Neither treatment protocol has any effect on other mitochondrial genes, such as ND2 (complex I) and ATPase 8 (complex V). Content of mRNA was normalized to 12S rRNA, a mitochondrial gene, as 12S rRNA expression did not change with miR-181c overexpresssion. *p<0.05 vs. sham (n=3).

**Figure S2. miR-181c Expression level in the Heart.** After 2 weeks of nanovector delivery we harevested the hearts and isolated the miRNA enriched fraction from the heart. qPCR data show that miR-181c expression is almost the same in the Plasmid miR-181c group compared to its sham group.
